# Effect of Atmospheric Pollutants on the Air Quality in Tunisia

**DOI:** 10.1100/2012/863528

**Published:** 2012-05-02

**Authors:** Karim Bouchlaghem, Blaise Nsom

**Affiliations:** ^1^Unité de recherche “Energétique et Environnement”, Institut Supérieur des Sciences Appliquées et de Technologie de Sousse, Université de Sousse, (03/UR 13-06), Cité Taffala, Ibn Khaldoun, 4003 Sousse, Tunisia; ^2^LBMS—EA 4325, Université de Bretagne Occidentale, Université Européenne de Bretagne, B.P. 93169, Rue de Kergoat, 29231 Brest Cedex 3, France

## Abstract

This paper presents the evolution of Saharan dust advection when the PM10 (particles with an aerodynamic diameter below 10 **μ**m) concentration exceeds standard limits in different Tunisian sites. Meteorological and concentration data (from 2004 to 2010) obtained from several monitoring stations and in situ measurements were used to identify African dust change in seasonal occurrence, their source origin, and their impact on surface PM10 concentrations. We pointed out that the Saharan dust contribution caused frequently the surpassing of the maximum number of days in excess of EU standard limits as well as of the maximum yearly average in the Mediterranean Tunisian coasts. The maximum daily concentration reaches 439 **μ**g/m^3^ during the Saharan events. The decrease in particulate levels recorded at the end of each event is due to the injection of European air masses and rainfalls. Primary pollutants peaks were much higher in winter than in summer which can be explained on the basis of the lower ventilation and mixing.

## 1. Introduction

Investigations of atmospheric aerosols (formation, transformation, and transport) and their contribution to the climate change and human health have become a primary topic in atmospheric pollution research. Health effects of aerosols are determined by their size distribution, chemical and microbiological concentration, and composition. The measurement of levels of atmospheric particulate matter (PM) is a key parameter in air quality monitoring across the world owing to the cause and effect relationship between exposure PM levels and health impacts (WHO, 2003) and their influence on the climate change. First, the atmospheric PM loadings are significant for climate change [1]. They may affect air temperatures through the absorption and scattering of solar radiation [2–5] and modifying cloud microphysical properties by acting as cloud condensation nuclei [6]. Furthermore, changes in atmospheric temperatures and in concentrations of condensation nuclei may affect convectional activity and cloud formation, thereby modifying rainfall amounts.

Second, a number of epidemiological studies have demonstrated that atmospheric pollution has a correlation with the daily deaths and hospitalisations as a consequence of pulmonary and cardiac disease responses [7–9].

Atmospheric PM10 is a multicomponent aerosol formed by anthropogenic and natural species. The natural particulate emissions are involved in heterogeneous reactions with anthropogenic gaseous pollutants and may modify the process leading to gas to particle conversion. Emissions of PM rise up in the air due to buoyancy effects, advect downwind, and disperse horizontally and vertically due to the turbulence field and prevailing meteorological patterns [7]. Due to its harmful effects on human health, PM10 is a subject of increasing concern. It may vary in their toxicological features [10]. To this end, exceeding the daily average of 50 *μ*g/m^3  ^is allowed for a maximum of 35 times per year, while the PM10 yearly average should not exceed 40 *μ*g/m^3  ^(EC, 1999). However, starting from January 2010, the maximum number of days exceeding 50 *μ*g/m^3  ^is lowered to 7 per year, and the year average is set at 20 *μ*g/m^3  ^(EU Directive 1999/30/CE).

Dust outbreaks may increase the ambient air levels of PM recorded in Tunisian air-quality monitoring networks due to the closed location of the Sahara resulting in exceeding the European Union (EU) daily limit of pollutants concentration. Furthermore, the low precipitation and the dry climate in the Mediterranean basin favours the long residence time of dust in the atmosphere [11, 12].

The occurrence of dust outbreaks affecting the Mediterranean regions is generally driven by intense cyclone generated south of Atlas Mountain by the thermal contrast of cold marine Atlantic air and warm continental air that cross North Africa during summer [13]. Therefore, it was important to investigate the ambient levels of PM10 concentration and the effects of Sahara dust events on the particulate matter.

Knowledge of the mechanisms that give rise to pollution episode in the Mediterranean regions is needed for the purpose of providing health advice to the public in events episodes. To this end, daily and seasonal variation of the main pollutants concentration and the meteorological conditions were studied in this paper.

The goal of this study is to present samples of the Saharan dust events and their impact on the spatial and temporal evolution of PM10 and air pollutants concentrations in the Tunisian sites and elaborating recommendations to competent organisms.

We study the Saharan dust transport and implications for the PM10 European standards limits and their influence on the annual, seasonal, and daily PM10 concentrations at several monitoring stations.

The studied regions and data sources are presented in Sections 2 and 3, respectively. Interesting Saharan events occurred in the Mediterranean Tunisian sites. They are studied in Section 4. The impact of air pollutants on the air quality is presented in Section 5.

## 2. Sites Description

**Figure 1:**
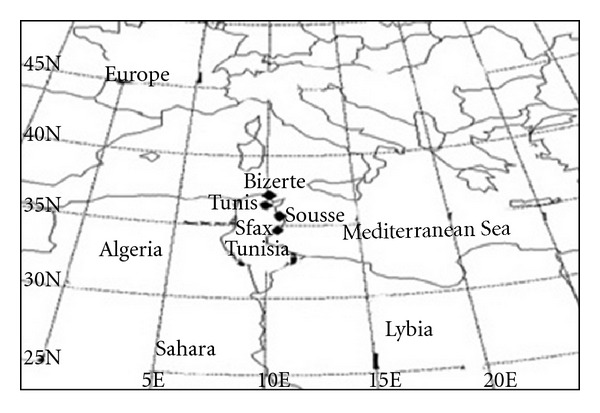
North African map displaying Tunisia, the measurement sites locations Sfax (34°44′N, 10°46′E), Bizerte (37°16′N, 9°52′E), Sousse (35°49′N, 10°38′), Tunis (36°49′N, 10°11′E), and the African Sahara.

Tunisia country is located in the North part of Africa (Figure 1). Its surface is 164.000 km^2  ^with 10 million inhabitants. Coastal cities share about 1300 km of beach and are widely influenced by the Mediterranean Sea. The sites presented in this study are Mediterranean cities with flat terrain.

Five industrial, residential, and urban monitoring stations Tunis (36°49′N, 10°11′E), Bizerte (37°16′N, 9°52′E), Sousse (35°49′N, 10°38′E), Sfax centre (34°44′N, 10°46′E), and its industrial site (34°44′N, 10°46′E) were selected for this study (Figure 1).

Bizerte city is located at the North part of Tunisia (37°16′N, 9°52′E). Its urban area accounts about 114.000 inhabitants. The measurement station sample is classified as urban which is mainly influenced by residential, traffic, and commercial activities.

Tunis City (capital of Tunisia) is also located in the North part of Tunisia (36°49′N, 10°11′E). The urban area (750.000 inhabitants) is about 212.63 km^2  ^surface. The sampling site is classified as urban, located in the vicinity of one of Tunis's major traffic avenues (Bab Saadoun Ave.).

Sousse city is located at the Eastern central part of Tunisia (35°49′N, 10°38′). The urban area (200.000 inhabitants) is about 45 km^2  ^surface. The sampling site is urban under the influence of residential, traffic, and commercial activities. The main industrial activities are a power plant and bricks work.

Finally, Sfax city is located at the south part of Tunisia (34°44′N, 10°46′E) with 270.000 inhabitants. The sampling site is industrial under the influence of intense chemical manufacturing activities.

## 3. Data and Methods

It might be highlighted that there is a lack of knowledge in Tunisia on the pollution concentration, since the national monitoring stations operated by the National Agency of Protection of Environment (NAPE) is localised in the most urban zones. All instantaneous concentrations data can be controlled from the central station.

All stations use standard NO*_x_* (NO and NO_2_),  O_3_, PM10, CO, and SO_2_ instruments designed by Teledyne Advanced Pollution Instrumentation Company (http://www.teledyne-api.com/). Data processing techniques and standard methods are described in the analyser instruction manuals. Used Teledyne models are 200A, 400A, and 100A for NO_*x*_, O_3_, and SO_2_, respectively. 

NO*_x_* Analyzer is designed to measure the concentration of nitric oxide NO, total oxides of nitrogen NO_*x*_, and, by calculation, nitrogen dioxide NO_2_. The instrument measures the light intensity of the chemiluminescent gas phase reaction of nitric oxide NO and ozone O_3_. The reaction of NO with ozone results in electronically excited NO_2_ molecules. The excited NO_2_ molecules release their excess energy by emitting a photon and dropping to a lower energy level. The light intensity produced is directly proportional to the NO concentration present.

The detection of ozone molecules is based on absorption of 254 nm UV light due to an internal electronic resonance of the O_3_ molecule. The Beer-Lambert equation calculates the concentration of ozone from the ratio of light intensities. SO_2_ Analyzer is based upon the technology from the measurement of fluorescence of SO_2_ due to absorption of UV energy. Sulfur Dioxide absorbs in the 190 nm–230 nm region. The UV lamp emits ultraviolet radiation and which passes through a 214 nm bandpass filter, excites the SO_2_ molecules, producing fluorescence. The fluorescent radiation is directly proportional to the concentration of SO_2_.

Levels of PM10 were calculated by means of automatic beta radiation attenuation monitors.

 Additionally, all stations were equipped with automatic weather monitoring. A mobile laboratory is used to control pollutants levels in several rural and urban sites.

The influence of atmospheric transport scenarios on the levels of Particulate Matters was investigated by means of back-trajectories analysis using the HYSPLIT Model (http://www.arl.noaa.gov/) and information obtained from TOMS-NASA, NRL aerosol and dust maps (TOMS, http://www.gsfc.nasa.gov/; NRL http://www.nrlmry.navy.mil/). Satellite images are provided by the NASA SEAWIFS project (http://seawifs.gsfc.nasa.gov/).

## 4. Contribution of Natural Events to Particulate Matter Concentration

**Figure 2:**
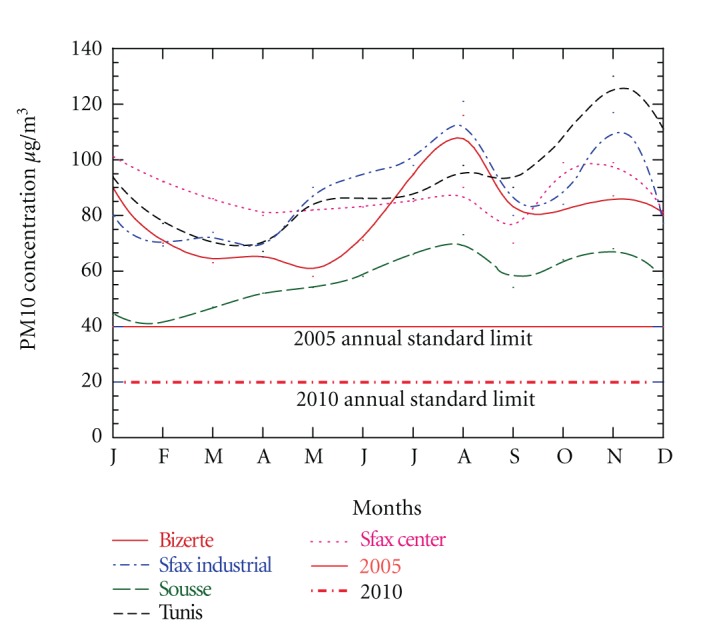
PM10 monthly averaged concentration recorded at the selected monitoring stations from January to December. (Solid and dashed lines are smooth curve fits).

Figure 2 pointed out that in the Mediterranean Tunisian regions the average seasonal evolution of PM10 is characterised by a winter maximum (November and December). The second maximum is observed during summer (July and August). The annual average PM10 concentration reached 58 *μ*g/m^3^ in Sousse, 80 *μ*g/m^3^ in Bizerte, 89 *μ*g/m^3^ in Sfax industrial site, 90 *μ*g/m^3^ in Tunis, and 87 *μ*g/m^3^ in Sfax center. These values are very high when compared to the PM10 annual limit of the 2010 EU Air Quality Directive (20 *μ*g/m^3^).

The satellite imageries and aerosol maps from (http://www.nrlmry.navy.mil/aerosol/) show that the Saharan events are present all year long during the study period.

**Figure 3:**
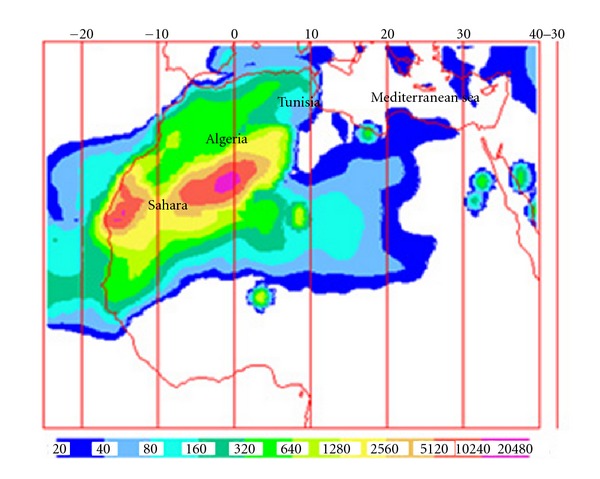
Evolution of the dust load from the NAAPS model (http://www.nrlmry.navy.mil/) during a sample of the Saharan events.

**Figure 4:**
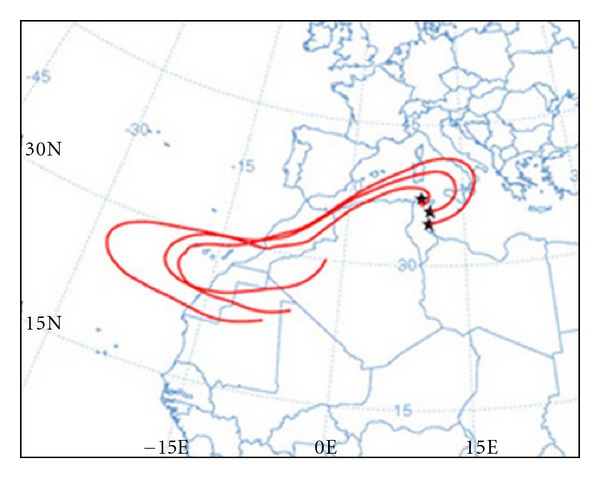
Sample of back-trajectories maps of Saharan event ending over the Mediterranean Tunisian regions at 1200 UTC. ((NOAA)'s Air Resources Laboratory (ARL)).

These Saharan events were produced by the North African high pressure which is centred over Algeria and/or Tunisia. First, the thermal convective activity over the Sahara induced by the heating of ground forces the injection of particles to high atmospheric levels. Second, the combination of both Atlantic cyclone and North African anticyclone systems characterising the North African weather is a scenario favouring the dust transport from the Sahara towards Tunisia and the Mediterranean Sea (Figure 3). Air back-trajectory and dust map of a sample of these transport scenarios are shown in Figure 4. Finally, the convective activity leads to the abatement of the Saharan air masses to the ground level. The mixing of the lower troposphere levels with upper atmospheric masses proceeding from North Africa during these intrusions is enhanced during summer by the greater thickness of the mixing layer due to the intense ground heating [14]. 

**Figure 5:**
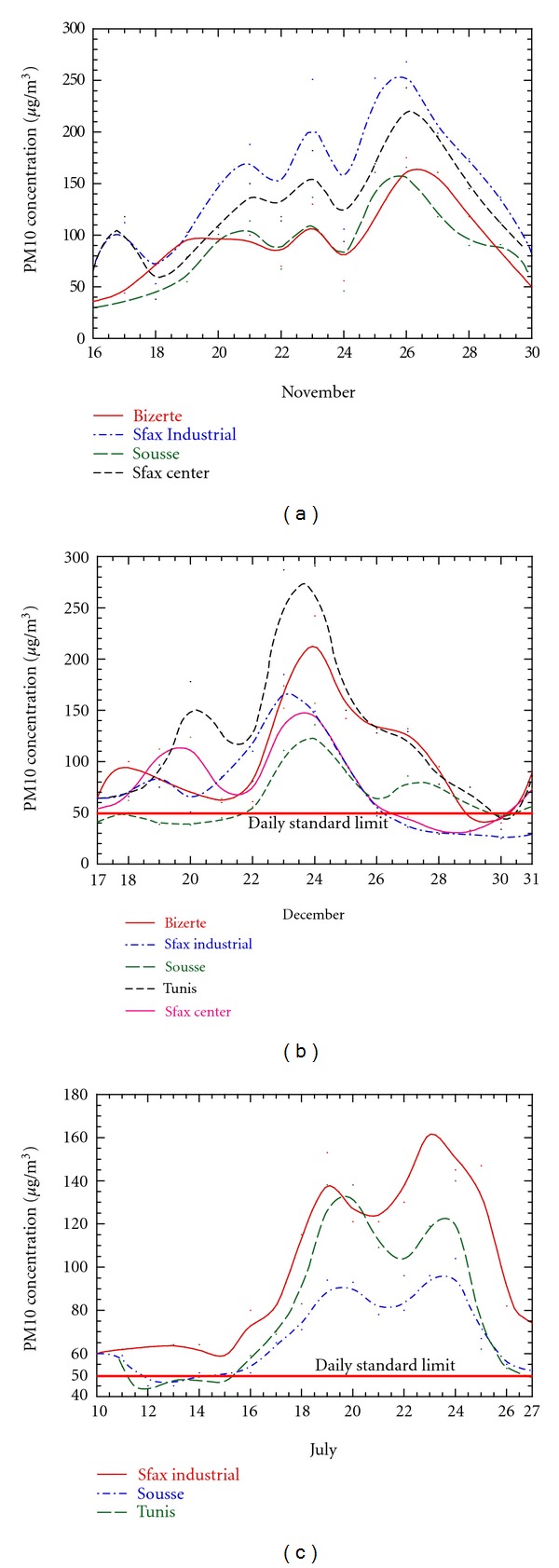
Concurrent daily PM10 average concentrations recorded at the selected monitoring stations over the study period along with identified exceeded values. (Solid and dashed lines are smooth curve fits).

These result in an increase in the daily PM10 concentrations at all the Tunisian air quality monitoring stations due to the high dust load of the Saharan air masses (Figure 5).

Figure 2 shows the daily PM10 levels recorded at the selected stations where the seasonal Saharan events are highlighted. For the sake of brevity, only some examples of the seasonal Saharan events will be discussed to point out the results of this study.

Figures 5(a) and 5(b) show the average daily variability of the PM10 concentrations during November and December, measured at the selected monitoring stations along with identified exceeded values. We can note the high degree of correlation between the PM10 concentrations recorded at the different sites. The PM10 daily levels exceeding the limit values of the EU standard were registered frequently at all the stations during these events (up to 300 *μ*g/m^3^). The data recorded during November and December were selected as a winter case study. The satellite imageries revealed the influence and intensification of the advection of a Saharan plume. Daily PM10 average levels of 166, 175, 209, 243, and 268 *μ*g/m^3^ were recorded in Sousse, Bizerte, Tunis, Sfax center, and Sfax industrial site, respectively, during these events (Figure 5(a)). The same figure shows the correlation between the PM10 concentrations recorded at the different stations. During the event of December a daily levels of 111, 242, 291, 149, and 157 *μ*g/m^3^ were recorded in Sousse, Bizerte, Tunis, Sfax center, and Sfax industrial site, respectively, (Figure 5(b)).

Before and after the events of Novembe,r the hourly average PM10 minimum reached 10, 4, 27, 23, and 25 *μ*g/m^3^ at Tunis, Bizerte, Sfax center, Sousse, and Sfax industrial stations, whereas during the event the maximum hourly PM10 concentrations reached 307, 378, 399, 130, and 439 *μ*g/m^3^, respectively. The back-trajectories (http://www.arl.noaa.gov/) and satellite images (http://www.nrlmry.navy.mil/aerosol/) show that low PM10 concentrations observed before the Saharan dust outbreak are caused by the presence of the North-West European air masses preceding the South-Westward particulate flow. The rapid increase in particulate levels is due to the plume behaviour of the Saharan intrusion. The decrease in particulate levels recorded at the end of the events is due to the arrival of European air masses and rainfalls. This is the origin of the Saharan red rains in Tunisia.

During the summer periods, the dust plume reached the Eastern Mediterranean Tunisian coast with the maximum increase in the PM10 concentrations on July. Daily average levels of 104, 140, and 179 *μ*g/m^3  ^were recorded in Sousse, Sfax industrial site, and Tunis stations, respectively (Figure 5(c)). We can note the high degree of correlation between the PM10 concentrations recorded at the different sites but at different levels due to the geographic location (the south sites are nearest to the Sahara) and characteristics of the monitoring stations (industrial, traffic, or residential).

The daily average PM10 concentrations measured at the selected sites during this period are high when compared with the 2010 EU limit values for PM10 concentrations (annual average of 20 *μ*g/m^3^, and do not exceed the daily concentrations of 50 *μ*g/m^3^ on more than 7 days/year, EU Directive 1999/30/CE).

For instance, in the urban station of Sousse, the PM10 annual average (58 *μ*g/m^3^) and the number of exceedances (188 days per year) of the limit value are higher than those of the European Directive. By looking to the origin of air masses using the aerosol maps, we remark that most of these exceedances may be attributed to Saharan events.

In the Sfax industrial station, both the PM10 annual mean (89 *μ*g/m^3^) and the number of exceedances (222 days per year) of the limit of the European Directive were largely surpassed. These values are very high when compared to the PM10 annual and daily limit values of the EU Air Quality Directive.

The homogeneity of the PM10 concentration and the number of daily exceedances obtained in the traffic (Tunis), urban (Bizerte and Sfax center), and industrial (Sfax) stations demonstrate the significance of the natural dust load in these regions.

The satellite imageries and aerosol maps from (http://www.nrlmry.navy.mil/aerosol/) show that the decrease in particulate levels after the events on July was induced by the eastward displacement of the Saharan dust toward the eastern Mediterranean and owing to the influence of the arrival of the European air masses. Furthermore, a common feature of a number of summer dust outbreaks caused by the North African dust transport is the slow reduction in the particulate levels after the Saharan event when compared with the winter events. This may be due to the following factors: first, the high convective dynamics account for a high resuspension of dust and for a slow renovation of the air masses, and second, the low atmospheric scavenging potential due to the low rainfall characterising the summer weather in the Mediterranean regions [15].

In addition to the high frequency of Saharan events in summer season, the PM10 peak may be related to the high photochemical conversion rate of gaseous pollutants to secondary aerosols (such as nitrates and sulphates), the high mineral dust load from soils induced by intense atmospheric convective dynamics and anthropogenic induced resuspension, the lower rainfall rate which reduces the particulate scavenging potential and an atmospheric particulate reservoir effect caused by a scarce renovation of the atmospheric masses in the Mediterranean basin [15].

Additionally, the temporal impact between the South and the North part of Tunisia is consistent with the geographical location of the monitoring stations with respect to the southwest origin of the Saharan event.

**Figure 6:**
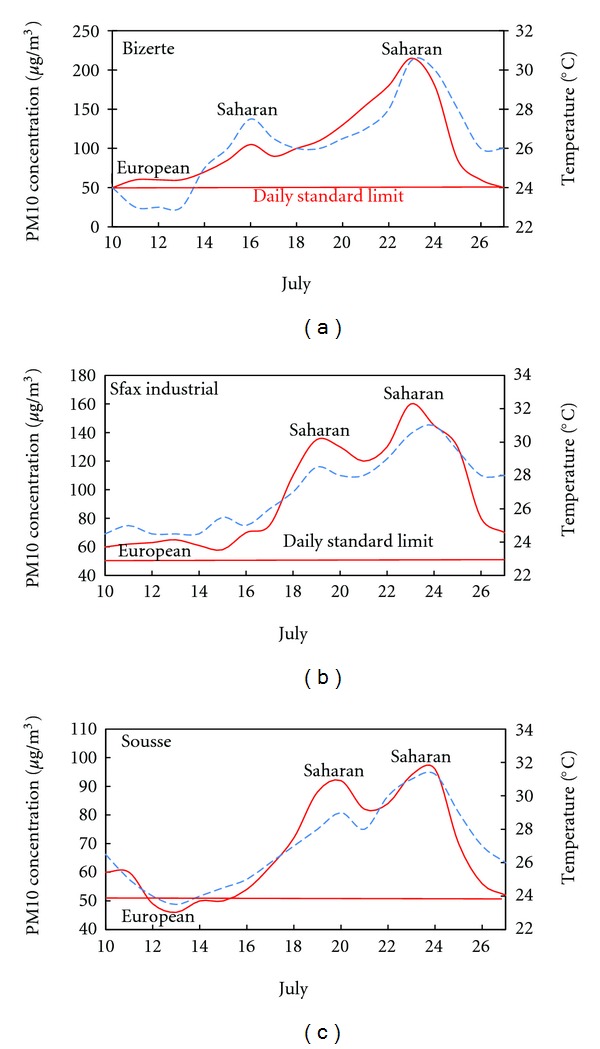
Daily variability of samples of the PM10 concentrations and temperature during summer in the selected monitoring stations. Time evolution of the left *y* axis is plotted with solid line, and the right one is plotted with dashed line. (Solid and dashed lines are smooth curve fits).

**Figure 7:**
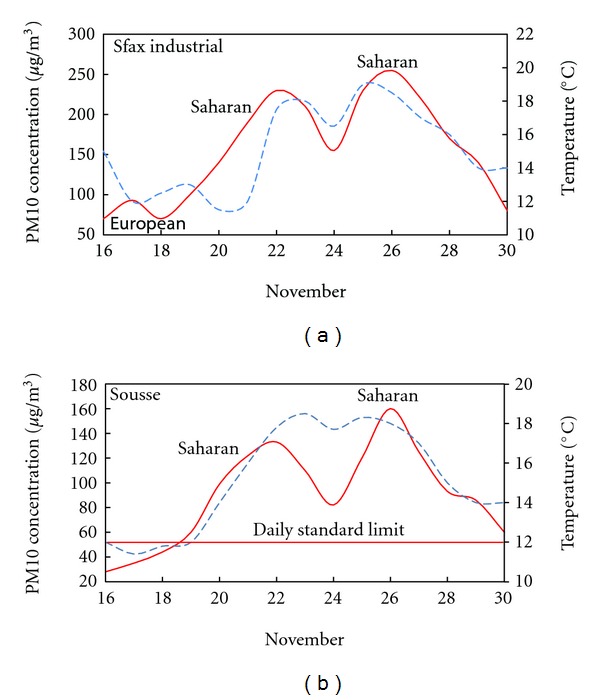
Daily variability of samples of the PM10 concentrations and temperature during winter in the selected monitoring stations. Time evolution of the left *y* axis is plotted with solid line, and the right one is plotted with dashed line. (Solid and dashed lines are smooth curve fits).

**Figure 8:**
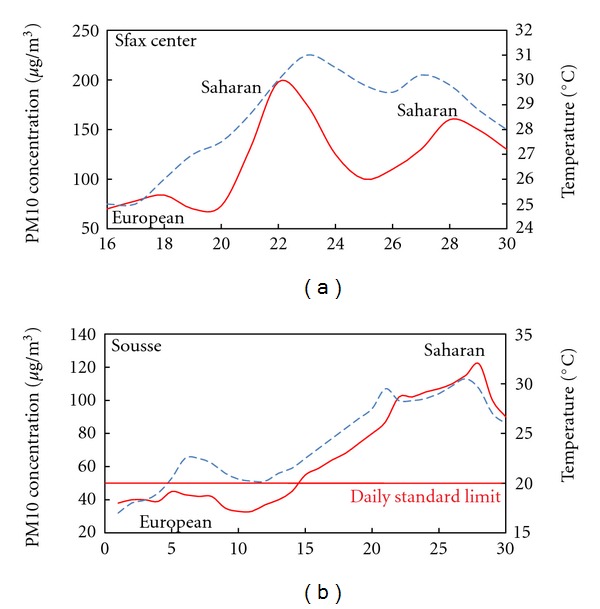
Daily variability of the PM10 concentrations and temperature during summer in the selected monitoring stations. Time evolution of the left *y* axis is plotted with solid line, and the right one is plotted with dashed line. (Solid and dashed lines are smooth curve fits).

Figures 6, 7, and 8 show the average daily variability of the PM10 concentrations and the temperature over different periods and seasons of the study period in the selected monitoring stations.

High temperatures were a common feature which coincides with an increase of PM10 concentrations. The maximum values were recorded between 1200 and 1400 Local Time (LT) (LT = UTC + 1 h), reaching 40°C on July. The temperature evolution was consistent with the PM10 concentrations. This may be due to the green house effect and the heating induced by the Saharan dust layer created over the selected sites during the events.

The correlation between PM10 concentration and temperature in different sites and seasons shows the origin of air masses. The high temperature and the high PM10 concentrations reveal that the air mass is of Saharan origin. The air mass loaded with dust instigates the increase of PM10 concentration at the surface. This is may be due to the formation of a dust layer over the studied sites. While the low temperature and decrease in PM10 concentration recorded before and after the events indicates the arrival of European air masses and rainfalls. The European episodes coincide with the decrease in the daily PM10 concentrations and temperature.

**Figure 9:**
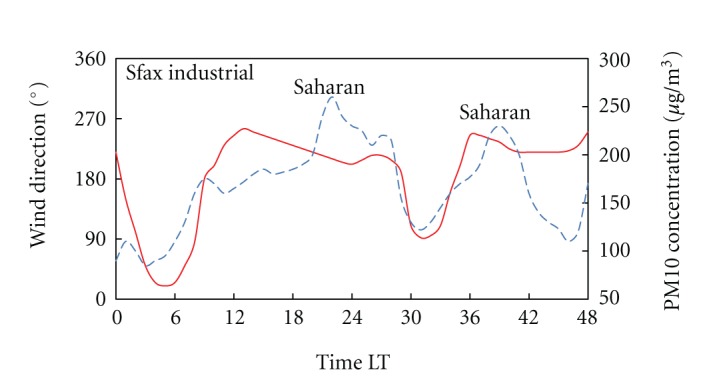
Time series plots of wind direction and PM10 concentrations during a Saharan dust sample period in Sfax's site. Time evolution of the left *y* axis is plotted with solid line, and the right one is plotted with dashed line. (Solid and dashed lines are smooth curve fits).

Figure 9 shows the hourly evolution of the average wind direction and the dust concentration during July in Sfax's site. The South and South-West trends are coincident with the spatial distribution of the African dust contribution to PM10 concentrations.

By looking at this figure, the wind direction daily cycle can be summarised as follows. During the period of cooling, from 00 to 0600–0700 LT, prevailing winds were North-East and East. As soil begins to heat up, the wind direction turns clockwise towards West and South-West and maintains this direction for the heating period. The hourly wind direction cycle was consistent with the thermal effects characterised by high values of PM10 concentrations, during the period of convective activity. This change of wind direction results in an increase of PM10 concentrations at all the monitoring stations. For instance we note that the South and South-West trends are coincident with the increase of the hourly PM10 concentration (up to 270 *μ*g/m^3^) in the monitoring station of Sfax during the events of July.

## 5. Effect of Air Pollutants on the Air Quality

**Figure 10:**
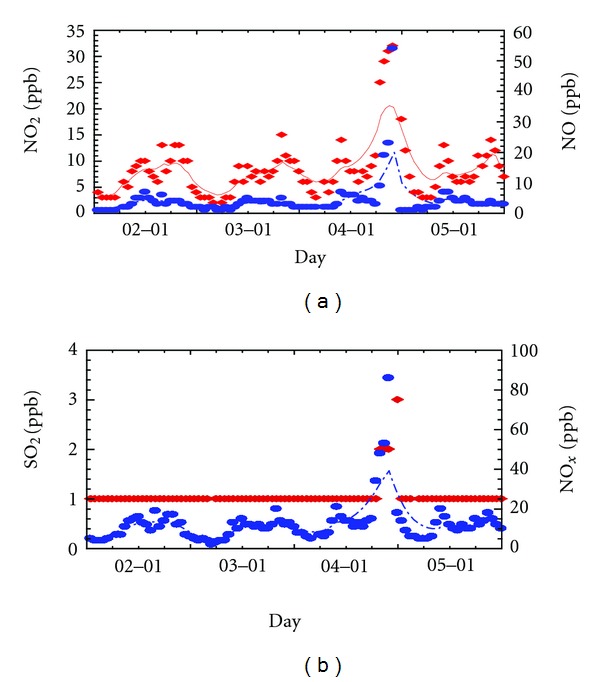
Hourly averaged series of pollutants concentrations during a winter period in Sousse city. Time evolution of the left *y* axis is plotted with solid line, and the right one is plotted with dashed line.

NO and NO_2_ peak is much higher in winter than in summer (up to 60 ppb). In spite of higher traffic in summer than in winter (national statistics have shown that, during the summer season, the vehicle number has doubled due to the increasing number of visitors), NO and NO_2  _higher peak in winter can be explained on the basis of lower ventilation and lower mixing (Figure 10).

NO, NO_2_, and NO*_x_* concentrations appear to be a common seasonal pattern across the sites. There is less air mixing in the lower boundary layer during the winter months, and this could lead to elevated levels of this pollutants. Additionally, the high winter concentration of NO_2_ could be enhanced by reduced photochemical activity of the reaction in which NO_2_ and (OH) radicals combine to form nitric acid (HNO_3_). The winter highs could also be linked to increase industrial and home heating.

With respect to the NO_2_, in winter, there is less O_3_ to oxidize the NO emissions, and the NO_2_, peak in the morning is hardly detectable. While by the end of the day, there has been sufficient buildup of O_3_ to oxidize some of the NO,  and a peak is detected during that period.

Simultaneously, NO, NO_2_, and SO_2_ increase to their maximum values showing evidence of low mixing and low ventilation effect during weak wind condition.

**Figure 11:**
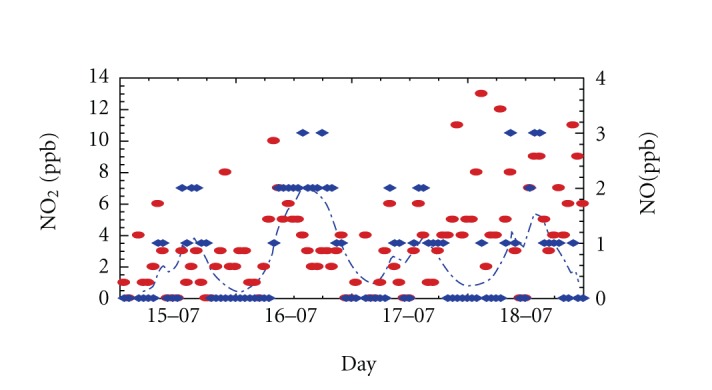
Hourly averaged series of pollutants concentrations during a summer period in Sousse city. Time evolution of the Left *y* axis is plotted with solid line, and the right one is plotted with dashed line.

The summer lows might be due to the enhanced photochemical activity on the presence of powerful solar radiation in which NO_2_ promotes ozone production. The early morning NO_2_ maximum does not necessarily coincide with that of NO (Figure 11). In fact, by this time, there is not enough O_3_ available for the oxidation to occur.

The O_3 _concentrations are much higher in summer (up to 65 ppb) than in winter (up to 35 ppb). During summer, meteorological conditions such as high temperature and thermal convection often induce the mixing of the air masses and the photochemical reactions. Observed ozone concentration may be the result of photochemical reaction of primary pollutants (NO*_x_* from traffic). Furthermore, the sea breeze also brings O_3_ [16], and the total concentration could result from a combination of local generation and regional transport.

## 6. Conclusion

This work shows that frequent Saharan intrusions reached the Mediterranean Tunisian regions due to its proximity to the African continent. The results highlighted the influence of the Saharan Dust Outbreak on PM10 concentrations recorded at several monitoring stations. The daily average PM10 concentrations increased in comparison to the levels recorded after and prior to the events. We note the correlation between the daily PM10 concentrations recorded at the different stations.

The impact of Saharan dust on the annual, seasonaln and daily PM10 levels is evident. An intensification of Southern heat waves is commonly associated with dust events in the Mediterranean Tunisian regions. We pointed out that at the end of these events the arrival of air masses from the European countries caused a decrease in PM10 concentrations and temperature that reaches values close to those obtained before the events. We show that the air masses loaded with dust instigate the increase of PM10 concentration and temperature at the surface. This may be due to the green house effect and heating created by the dust layer over the Mediterranean Tunisian sites during the Saharan events. In winter season, primary pollutants peaks were much higher than in summer which can be explained on the basis of lower ventilation in the winter and lower mixing.
